# Activating Progressive Sn^2+^ Nucleation by Micellar Structure Electrolyte for Dead-Sn-Free Aqueous Batteries

**DOI:** 10.1007/s40820-026-02070-6

**Published:** 2026-02-03

**Authors:** Xiaojia Lan, Zhaoyu Zhang, Yuekai Lin, Wencheng Du, Yufei Zhang, Minghui Ye, Zhipeng Wen, Yongchao Tang, Xiaoqing Liu, Cheng Chao Li

**Affiliations:** 1 School of Chemical Engineering and Light Industry, Guangdong University of Technology, Guangzhou, 510006 People’s Republic of China; 2 Guangdong Provincial Laboratory of Chemistry and Fine Chemical Engineering Jieyang Center, Jieyang, 515200 People’s Republic of China

**Keywords:** Aqueous electrolyte, Sn anode, Dead Sn, Micellar electrolyte, Progressive nucleation

## Abstract

The instantaneous nucleation of Sn originating from the uncontrolled diffusion of Sn^2+^ ions typically forms large, electrochemically inactive “dead Sn” that severely constraints the plating/stripping reversibility of Sn anode for acidic aqueous batteries. Herein, nanoscale spatial confinement of Sn^2+^ ions is realized in SnSO_4_ electrolyte by strategically dictating spontaneous assembly of nanomicelles with amphipathic sulfolane. The as-constructed locally heterogeneous environment ensures the sustainable release of Sn^2+^ ions, which reprograms the nucleation manner from instantaneous to progressive modes. The consequent progressive formation of Sn nuclei triggers size refinement of electrodeposited Sn, thereby alleviating the “dead Sn” issue. Meanwhile, the reaction competitivity of Sn^2+^ reduction over hydrogen evolution side reaction is effectively strengthened as the consecutive hydrogen bonding network among bulk water is disrupted by the micellar structure. Consequently, Sn anode exerts an unprecedently high average Coulombic efficiency of 99.97% and witnesses a prominent life span extension from 710 to 8400 h (~ 11-fold enhancement). In a dual-plating configuration, the Sn||Mn full battery delivers a 1.6 V discharge plateau and sustains 790 cycles, demonstrating practical feasibility. Our findings underscore the decisive role of the very initial nucleation behavior in regulating metal electrochemistry, applicable to other multivalent anodes.

**Supplementary Information:**

The online version contains supplementary material available at 10.1007/s40820-026-02070-6.

## Highlights

- Nanoscale spatial confinement of Sn^2+^ ions is realized in SnSO_4_ electrolyte by strategically dictating spontaneous assembly of nanomicelles with amphipathic sulfolane.
- The sustained release of Sn^2+^ ions reprograms nucleation from an instantaneous mode to a progressive mode, resulting in a Dead-Sn-free deposition layer that significantly extends the Sn anode life span from 710 to 8400 h.
- In a dual-electrode-free configuration, the Sn||Mn full battery delivers a high-discharge-voltage plateau at ~1.6 V and exerts stable cycling over 790 cycles.

**Supplementary Information:**

The online version contains supplementary material available at 10.1007/s40820-026-02070-6.

## Graphical abstract

**Figure:**
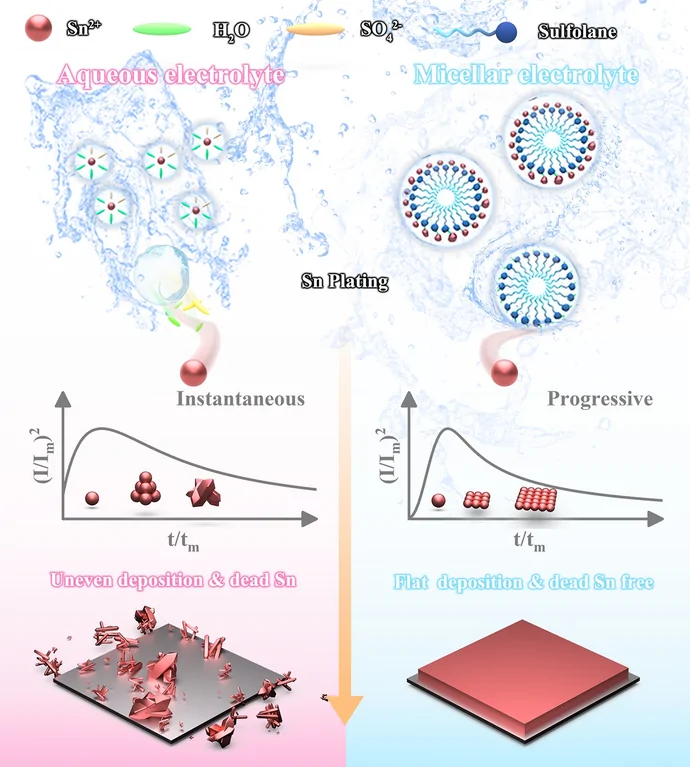

## Introduction

The ever-growing demand for sustainable renewable energies like wind or solar, in view of their intermittency and fluctuation feature, has driven the rapid development of cost-effective and application-tailored technologies such as secondary batteries [1, 2]. The currently most extensively studied lithium-ion batteries, due to their unparalleled high energy density, have been widely applied in mobile phones, computers, electric vehicles and other electric fields [3]. However, the high reactivity of lithium metal and the toxicity of flammable organic electrolytes have led to frequent safety incidents, arousing rigorous concerns about their widespread future applications in power plants or uninterrupted power systems [4]. Therefore, developing alternative rechargeable batteries embedding intrinsic high-safety characteristic has become an urgent priority, especially those adapted to large-scale energy storage. Against this backdrop, nonflammable acidic aqueous batteries (AABs) based on metal anodes have gained increasing research attention. Metals commonly used in AABs such as Zn, Fe and Pb, are highly attractive due to their potential for high energy density [5]. However, the weakly acidic feature of aqueous electrolytes typically employed for Zn and Fe (pH = 4–6) substantially limits their cycling durability as hydrogen evolution reactions (HER) and side reactions inevitably take place [6, 7]. Additionally, dendrite formation poses a significant challenge for these metal anodes. Metal dendrites with high Young's modulus (200 GPa of Fe and 108 GPa of Zn) can easily pierce the separator, causing rapid battery failure [8]. Although Pb metal witnesses long-term stability even under strongly acidic conditions (e.g., 2 M H_2_SO_4_), its high toxicity contradicts the fundamental safety goal of aqueous batteries [9]. Therefore, it is imperative to explore potential metal anode candidates embracing high stability and environmental friendliness in aqueous environments.

Recently, Sn metal emerges as a standout candidate among various metal anodes for AABs due to its remarkable advantages [10, 11], including high theoretical specific capacity (451.6 mAh g^−1^, based on Sn^2+^/Sn), distinct anti-HER properties and low Young's modulus (42 GPa). Furthermore, its non-toxic nature, as demonstrated by its widespread application in food packaging industry, aligns perfectly with the requirements for high safety and environmental friendliness. Despite these fascinating merits, when working as a metallic anode in strongly acidic environment, Sn suffers from poor plating/stripping reversibility due to the ubiquitous occurrence of “dead Sn” and the imperceptible hydrogen evolution [12]. Specifically, the intrinsic body-centered tetragonal structure of Sn endows different crystal planes with minimal surface energy differences. The resultant isotropic growth during the plating course facilitates the formation of large Sn particles (diameter > 50 µm) or long Sn whiskers (~ mm scale) that are inclined to peel off from the electrode surface as “dead Sn” due to the fragile solid–solid contact. In addition, though their low redox potential (− 0.14 V vs. standard hydrogen electrode (SHE)), the scattered, isolated Sn deposits remaining on the electrode, especially their sharp edges and corners exposed to concentrated acidic electrolyte containing 1–3 M H_2_SO_4_, show poor competitiveness over hydrogen evolution and thus undergo severe electrolyte corrosion. The hydrogen gas release in turn intensifies the detachment of fresh Sn deposits from the substrate. The low Coulombic efficiency (CE) attributed to these two close-loop courses are now the main constraint for reversible Sn chemistry.

To minimize the negative impact of “dead Sn” and associated HER, numerous research efforts have been devoted to modulate the deposition morphology of Sn in acidic aqueous electrolytes, by either substrate modification or electrolyte formulation. For instance, leveraging the interfacial alloying effect between Cu and Sn, Lu et al. introduced Cu metal as a stannophilic deposition substrate to achieve Sn deposits with smaller particle size and more uniform distribution, enabling stable cycling for over 260 h [12]. In terms of electrolyte engineering, Xia et al. constructed a stable Sn anode with remarkable cycling stability up to 1000 h by introducing gelatin additive into SnSO_4_ to regulate the interfacial adsorption species [13]. Similar life extension effect can also be attained by finely designing the solvation structure of Sn^2+^ [14, 15]. Apart from such inspiring advances in Sn stabilization, the long-term durability of Sn anode under harsh test conditions (either > 1 mAh cm^−2^ or > 10 mA cm^−2^) is still challenging because the detrimental “dead Sn” issue would become substantially intensified with the increase in the test current density or the utilization rate of Sn metal. More importantly, the underlying mechanism for the formation of “dead Sn” has been seldom discussed and remains mysterious. Indeed, Xia et al. did notify the unique contribution of the high exchange current density (I_0_) of Sn^2+^/Sn to the undesired generation of large-size Sn particles and realized stable Sn plating/stripping over 300 h at 10 mA cm^−2^, 1 mAh cm^−2^ by increasing the desolvation penalty of Sn^2+^ complexes with methanesulfonate anions. Nevertheless, the vital role of the initial nucleation behavior of Sn deposits, a critical fundamental foundation of subsequent crystal growth and final Sn morphology, is generally neglected.

Pure aqueous electrolytes are generally considered as macroscopically homogeneous ionic transport media in spite of their complex chemical composition and atomistic solvation structures. In this work, amphipathic sulfolane embedding a polar tinophilic “head” attached to a tinophobic “tail” is strategically introduced into 0.2 M aqueous SnSO_4_ electrolyte (BE) to initiate the spontaneous assembly of nanomicelles encapsulated with Sn^2+^ ions. Such locally heterogeneous environment constructed by the as-designed micellar structure electrolyte (MCE) transits the nucleation manner of electrodeposited Sn from instantaneous (refers to the formation of nuclei on the electrode surface only at an instance, leading to coarse, irreversible Sn deposits) to progressive (yielding finely dispersed, electrochemically active Sn nuclei) process. Specifically, as Sn^2+^ ions are overwhelmingly trapped in the nanomicelles, the release of Sn^2+^ ions during the charge course is inevitably retarded. The consequent progressive formation of Sn nuclei triggers size refinement of electrodeposited Sn, thereby alleviating the “dead Sn” issue in sulfolane-modified electrolyte. Furthermore, in MCE, the original consecutive hydrogen bonding network of water is partially disrupted by the localized intermolecular hydrogen bonding effect between sulfone and water molecules, thereby suppressing the occurrence of HER originating from uninterrupted proton migration. Accordingly, the cycling durability of Sn||Sn symmetric cell is substantially prolonged from 710 h to more than 8400 h at 5 mA cm^−2^ and 1 mAh cm^−2^, accompanied by an unprecedently high average CE up to 99.97%. Similar prominent life span extension of Sn anode brought by the micellar-mediated nucleation manner transition is also testified in MCE at harsh test conditions, in terms of both high current density (20 mA cm^−2^, ~ 700 h) and large areal capacity (4 mAh cm^−2^, ~ 380 h) where BE can hardly afford. In addition, the superiority of the micelle electrolyte is further validated in the Sn||Mn electrolytic full batteries. Even under dual-electrode-free configuration, the device exerts stable cycling over 790 cycles, along with a high-discharge-voltage plateau at ~ 1.6 V. This work highlights the decisive role of the very initial nucleation behavior in regulating the reversible Sn chemistry, which is to deepen current understanding on the superior performance of metal anodes commonly visualized in water/organic mixed electrolytes.

## Experimental Section

### Chemicals and Materials

Tin sulfate (SnSO_4_, AR), manganese sulfate (MnSO_4,_ AR), sulfolane (AR) and sulfuric acid (H_2_SO_4_) were purchased from Aladdin. Sn foil (99.95%), Cu foil (99.95%) and graphite paper (50 μm) were purchased from Canrd Technology Co. Ltd. All these chemicals were used as received without further purification.

### Preparation of Electrolytes

The electrolytes were prepared by dissolving SnSO_4_ and sulfolane into 2 M H_2_SO_4_ aqueous solution. We prepared five kinds of electrolytes including pure 2 M H_2_SO_4_ + 0.2 M SnSO_4_ without and with 5, 10, 15 and 20 v/v% sulfolane (denoted as BE, MCE-1, MCE-2, MCE-3 and MCE-4 accordingly), for use. Unless stated, the electrolyte denoted as MCE in the manuscript refers to the sample supplemented with 10% sulfolane.

### Electrochemical Measurements

The cyclic voltammetry (CV) curves, linear sweep voltammetry (LSV) curves, linear polarization measurements, electrochemical impedance spectroscopy (EIS) and Tafel test were obtained on Gamry electrochemical station. For the CV of Sn^2+^/Sn chemistry and LSV, Cu foil was used as the working electrode, Sn foil was used as the counter electrode, and a Hg/Hg_2_SO_4_ electrode was used as the reference electrode, with a scan rate of 5 mV s^−1^. Tafel test used Sn foil as working electrode, Pt as counter electrode and mercury–mercurous sulfate electrode as reference electrode. Exchange current density (I_0_) was measured by linear polarization curves with a scan rate of 0.1 mV s^−1^ from − 5 to 5 mV vs. open-circuit potential (OCP). The current transient curve was measured using chronoamperometry in a three-electrode system, with a glassy carbon working electrode, Pt counter electrode and Ag/AgCl reference electrode. The experiment was conducted through stepwise potential increments. The raw curve is reported to identify the maximum point and subsequently plot the dimensionless curve of (*I*/*I*_m_)^2^ versus *t*/*t*_m_, where *I*_m_ and *t*_m_ represent the maximum current density and its corresponding time, respectively. The dimensionless curves were fitted with theoretical instantaneous and progressive nucleation curves described by the Scharifker–Hills’ models (see details in the manuscript). Sn||Cu and Sn||Sn symmetric cells were assembled to evaluate the CE and cycling durability of Sn anodes in different electrolytes using CR2032 coin-type cells. The Sn||Mn battery was tested in a two-electrode system in the 10 mL electrolyte. For the pouch cell assembly, all the electrodes (3 cm × 3 cm) were the same as the previous tests. The electrolyte injected into the cell was ≈4 mL. The assembled pouch battery was encapsulated in an aluminum–plastic film by the vacuum sealer. The galvanostatic discharge/charge tests were measured on a NEWARE Battery Testing System (CT-4008-5V6A-S1-F, Shenzhen, China) at room temperature.

Exchange current density (*I*_0_) can be calculated from the slopes of linear polarization curves, based on the

1 $${I}_{0}=\frac{{{IRT}}}{F\eta }$$

where *I*, *F* and *η* signify the applied current density (A cm^−2^), the Faraday constant (96,500 C mol^−1^) and the total overpotential (V), respectively.

The activation energy (*E*_a_) can be evaluated according to the Arrhenius equation:

2 $$\frac{1}{{R}_{\rm ct}}=A\mathrm{exp}\left(-\frac{{E}_{\mathrm{act}}}{RT}\right)$$

where *R* and *T* signify the gas constant (8.314 J mol^–1^ K^–1^) and the temperature (K), respectively.

## Results and Discussion

**Fig. 1:**
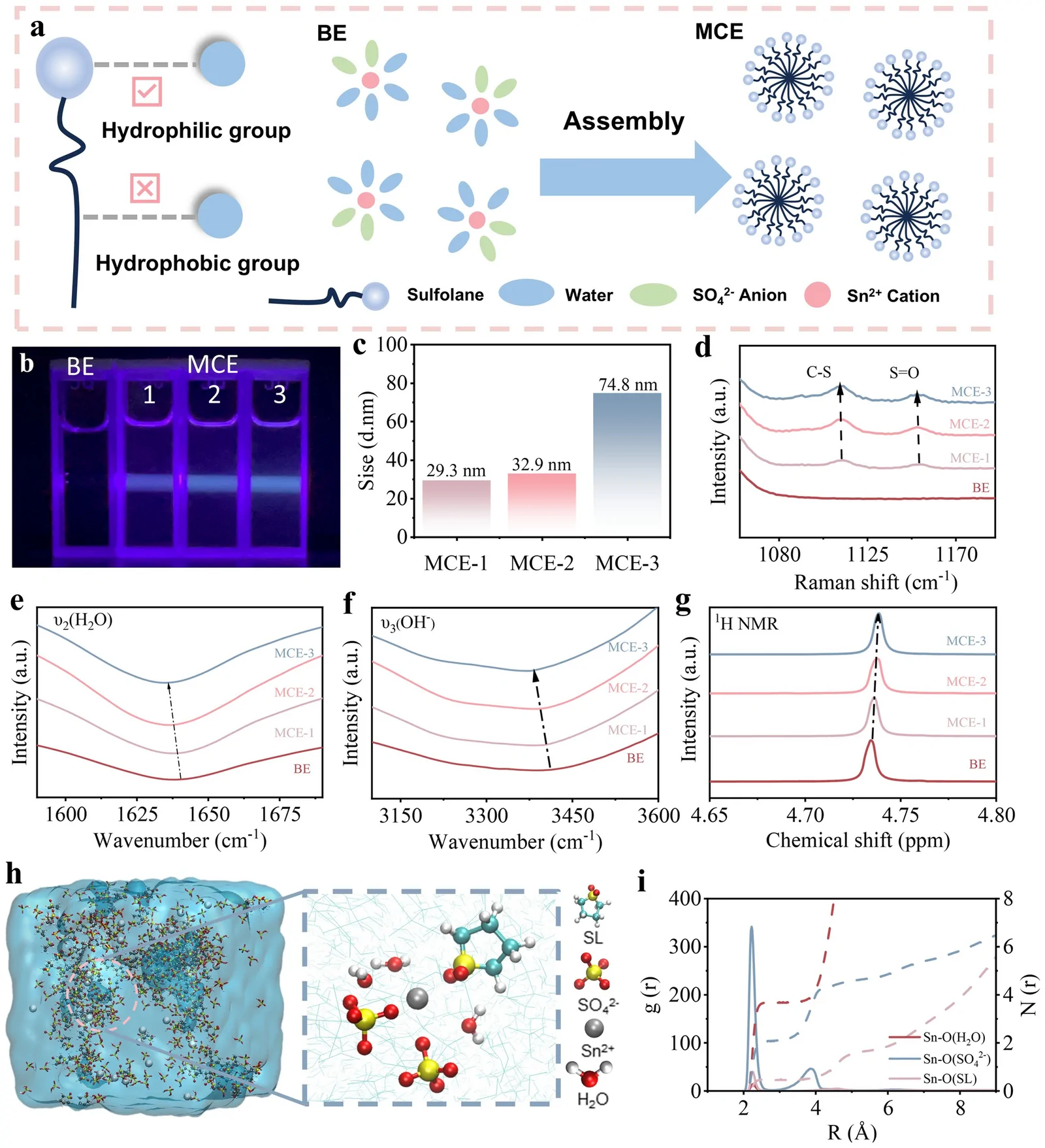
**a** Schematic diagram of amphiphilic characterization of sulfone and the micellar electrolyte self-assembly in MCE samples. **b** Tyndall effect investigation of BE, MCE-1, MCE-2 and MCE-3. **c** Summary of DLS analysis of micelle size distribution. **d** Raman, **e** and **f** FT-IR, **g** ^1^H NMR spectra of BE, MCE-1, MCE-2 and MCE-3. **h** 3D snapshots of MD simulations for MCE with corresponding schematic diagrams of Sn^2+^ solvation structures. **i** RDF of Sn–O (H_2_O), Sn–O (SO_4_^2−^) and Sn–O (SL) in MCE and the corresponding coordination numbers

In this study, 0.2 M SnSO_4_ was dissolved into 2 M H_2_SO_4_ to prepare the benchmark electrolyte (BE). Sulfolane, a versatile dipolar aprotic solvent featuring unique amphipathic characteristics attributed to its strong hydrophilic (–S=O) and hydrophobic groups (–CH_2_), is selected as a functional additive to initiate spontaneous self-assembly of micellar aggregates in aqueous saline electrolyte [16]. The purpose is to create microscopic heterogeneous environment to transform the initial nucleation behavior of Sn^2+^ ions from instantaneous to progressive and thereby modulate the subsequent crystal growth of Sn. This small organic molecule displays high stability against strong acids and is capable of strong solvation of Sn^2+^ cations by the electron-rich oxygens in the –S=O group [17]. As illustrated in Fig. S1, sulfolane is miscible with BE, but the salting out of SnSO_4_ is noticed when its volume concentration reaches 20%. Therefore, in addition to BE, we prepared three kinds of clear and macroscopically homogeneous, transparent electrolyte mixtures, BE with 5, 10 and 15 v/v% sulfolane for further comparison (denoted as MCE-1, MCE-2 and MCE-3 in order, accordingly). The electrostatic potential (ESP) maps of sulfolane and H_2_O (Fig. S2) reveal that the –S=O group is more attractive to electron-deficient species (hydrogen atoms, protons and Sn^2+^ ions) due to a more negative charge (− 0.576 eV) distribution than O–H in H_2_O (− 0.385 eV). The lower binding energy of sulfolane–H_2_O compared to H_2_O–H_2_O (− 0.21 eV *vs.* − 0.30 eV, Fig. S3) by density functional theory (DFT) calculations further highlights this characteristic. Therefore, –S=O groups are anticipated to form stronger intermolecular hydrogen bonds with H_2_O through intense dipole–dipole interactions while hydrophobic -CH_2_ groups are to induce topological defects in the tetrahedral hydrogen bonding network of the host water [18]. With the disruption of original continuous hydrogen bonding network in H_2_O, spontaneous self-assembly of micellar aggregates is expected in the three MCE samples (Fig. 1a). To verify our hypothesis, vertical irradiation of BE and MCEs by a violet laser pointer is performed. Distinct from the macroscopically homogeneous feature of the BE counterpart, the presence of sulfolane invariably triggers strong typical Tyndall effect despite its volume ratio, proving the emergence of stable micelle aggregates in MCE-1, MCE-2 and MCE-3 (Fig. 1b). The size distribution of the as-formed micelle structure in the three samples investigated by dynamic light scattering (DLS) studies (Figs. S4 and 1c) discloses that the micelle size in MCE-2 is slightly larger than that in MCE-1 (32.9 and 29.3 nm, respectively). A sharp micelle size rise to 74.8 nm is visualized in MCE-3, which may be attributed to the severe sulfolane aggregation. The results of the above two experiments firmly evidence the sulfolane clustering phenomenon in these three MCE samples.

We then investigated the specific impact of sulfone-involved micellar electrolyte on the Sn^2+^ solvation structure and hydrogen bonding network of water, two key parameters decisive to the plating/stripping efficacy of Sn anode in aqueous electrolytes [15]. Raman spectroscopy and Fourier transform infrared spectroscopy (FT-IR) were used to unravel the solvation structure disparities of Sn^2+^ in BE with and without sulfone. According to Raman spectra, in contrast to BE, two characteristic peaks attributed to C–S and S=O groups appear at around 1080 to 1170 cm^−1^ upon the presence of sulfolane (Fig. 1d). With the gradual rise of sulfolane ratio, both peaks experience continuous a red shift, which may be initiated by its varied self-assembly degree into micellar structures [19]. Specifically, the ordered alignment of sulfolane's dipoles within the micellar corona creates cooperative electric fields through constructive superposition of individual dipole moments. The resultant enhancement of magnetic field induces a red shift in the vibrational frequencies of C-S and S=O bonds via electrical interactions. Such enhancement of the local electric field may also be conducive to the entrance of sulfolane into the solvation structure of Sn^2+^. FT-IR characterization further confirms the solvation structure change of Sn^2+^ initiated by sulfolane (Fig. 1e, f). The strong peaks observed in the ranges of 2980–3630 and 1550–1700 cm^−1^ are attributed to the O–H stretching (ν_3_ (O–H)) and bending vibrational (ν_2_ (H_2_O)) modes of H_2_O molecules, respectively. With the concentration rise of sulfolane from 5 to 15% v/v, the characteristic peak attributes to ν_2_ (H_2_O) undergoes slight red shift from 1639.5 to 1636.4 cm^−1^. Similar trend is also observed from ν_3_ (O–H), implying the H_2_O solvated to Sn^2+^ may be replaced by sulfolane. As the strong electrophilicity of sulfolane facilitates its intense interaction with Sn^2+^, the electrostatic attraction between Sn^2+^ and SO_4_^2−^ is weakened, as evidenced by peak shift of the stretching mode signal of SO_4_^2−^ groups from 981.1 to 976.4 cm^−1^ in Raman spectra (Fig. S5). It can thus be deduced that with the presence of micellar structure in MCE, the solvation structure of Sn^2+^ is altered, *i.e.,* the replacement of H_2_O by sulfolane in the solvation sheath. Specifically, dissociated Sn^2+^ ions are readily coordinated with Sn philic S=O “head” in MCE. In addition to affecting H_2_O in the solvated structure, the hydrogen bonding effect between H_2_O molecules in the bulk phase can also be broken by the nanoscale micelles. The O–H stretching vibration signal in Raman spectroscopy can be further deconvoluted into the contribution of strong (3230.2 cm^−1^), medium (3417.9 cm^−1^) and weak (3552.7 cm^−1^) hydrogen bonds [20]. As illustrated in Fig. S6, the percentage of strong H bond ratios substantially increases attributed to the intense intermolecular hydrogen bonding interaction between sulfolane and water. Similar conclusion can also be drawn from the ^1^H chemical shift increases from 4.725 to 4.741 ppm in the ^1^H nuclear magnetic resonance spectroscopy (NMR) (Fig. 1g). That is, the micellar architecture can disrupt Grotthuss-type proton hopping pathways within the aqueous phase, which effectively suppresses the activity of both free water molecules and hydrated protons and thereby inhibiting relevant HER [6].

Molecular dynamics (MD) simulations were further employed to disclose the solvation structure evolution of Sn^2+^ in BE and MCE. Figures 1h and S7 present the 3D MD snapshot of the solvation structure of Sn^2+^ in the two systems. The statistical results show that, in BE, on average, one Sn^2+^ is coordinated with four H_2_O molecules and two SO_4_^2−^ anions. In MCE, the coordination number (CN) of H_2_O is reduced from 4 to 3, which is in good agreement with our previous spectroscopic characterization results that sulfolane is involved in the solvation shell of Sn^2+^.The precise transition of the Sn^2+^ coordination structure of is further confirmed by the Radial distribution function (RDF) and CN analyses. As depicted in Fig. S8, in BE, the sharp peak located at 2.25 Å is contributed by Sn-OH and Sn-SO_4_^2−^ in the first solvation shell of Sn^2+^, with CNs of 4.2 and 2, respectively. In MCE, owing to the presence of sulfolane, a coordination peak corresponding to the Sn–sulfolane (Fig. 1i) is also noticed at 2.25 Å (CN ~ 0.5). As the CN of Sn-SO_4_^2−^ remains the same (~ 2) in the two systems, the involvement of sulfolane in the solvation structure of Sn^2+^ is mainly achieved by repelling H_2_O out of the first solvation sheath, as evidenced by the CN decrease of Sn-OH from 4.2 to 3.6. The substitution of H_2_O by sulfolane in the solvation sheath is expected to effectively weaken the hydrogen evolution side reaction [21]. In addition, due to the large steric hindrance of the micellar structure, the desolvation penalty of sulfolane–Sn^2+^ complex would be augmented, thereby reducing the migration rate of Sn^2+^ ions [13]. Mean square displacement (MSD) of Sn^2+^ further validates this hypothesis (Fig. S9). Specifically, the Sn^2+^ diffusion coefficient significantly decreases from 0.63 to 0.17 nm^2^ ns^−1^, indicative of the unique Sn^2+^-encapsulation capability of the micellar structure. The retarded Sn^2+^ transfer is expected to shift the nucleation behavior of Sn^2+^ ions from instantaneous to progressive, restrict the uncontrolled rampant deposition of Sn^2+^, thereby prevent the occurrence of “whisker growth” and “dead Sn.” Preliminary concentration screening was conducted via electrochemical testing (see Fig. S10 for results). Notably, the battery's cycle life initially increased then decreased with rising sulfolane concentration, likely due to excessively large micellar aggregates of MCE-3. Consequently, MCE-2 was confirmed as the optimal concentration for reversible tin chemical reactions. For subsequent discussion, it will be referred to as MCE.

**Fig. 2:**
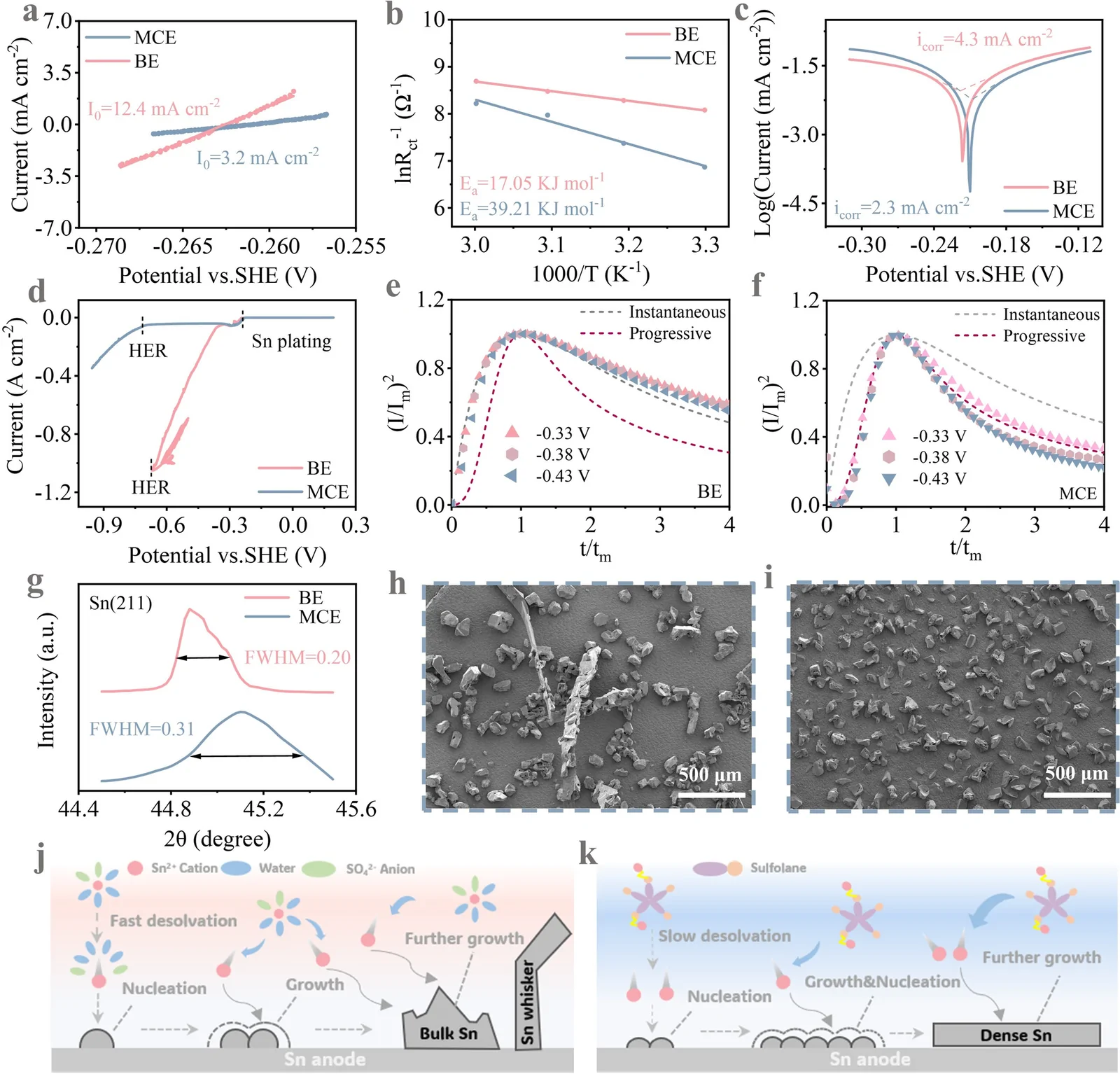
**a** Exchange current densities of Sn electrode in BE and MCE. **b** Activation energies of Sn^2+^ in BE and MCE. **c** Tafel and **d** LSV curves of Sn anodes in BE and MCE. Dimensionless current–time transients of **e** BE and **f** MCE at different potentials compared with the classical instantaneous and progressive nucleation models. **g** Enlarged XRD partial view of the Sn (211) facet. SEM images of Sn deposition morphology on Cu substrate in **h** BE and **i** MCE with an areal capacity of 1 mAh cm^−2^. Schematic illustration of nucleation and growth mechanism after desolvation of Sn^2+^ in **j** BE and **k** MCE

To verify stable is the micellar structure under high current densities and prolonged cycling, we measured the dynamic light scattering (DLS) of the electrolyte after 100 cycles at a high current density of 5 mA cm^−2^ (Fig. S11). Similar peaks (~ 34 nm) remain observable, indicating that the assembled micelles retain excellent electrochemical stability even after high-current operation and extended cycling. During long-term cycling at high current densities, changes in electrolyte ion concentration and environmental alterations may cause minor perturbations to the micelle structure, yet its fundamental properties remain unchanged. The electrochemical behaviors of metallic Sn foil in BE and MCE were studied to highlight the influence of micellar electrolyte structure on Sn plating/stripping chemistry. In MCE, as Sn^2+^ ions are confined within the micellar structure, the desolvation course is anticipated to be impeded, thereby slowing down the electrochemical kinetics [22]. As evidence, the exchange current density (*I*_0_), a common kinetic indicator of the deposition/dissolution course of Sn^2+^/Sn, is reduced from 12.4 to 3.2 mA cm^−2^ (Fig. 2a). The desolvation activation energies (*E*_a_) of Sn^2+^ in the two electrolytes were then calculated based on the EIS data of Sn||Sn symmetric cells (Fig. S12) according to the Arrhenius equation. Consistent with the reduction of *I*_0_, the *E*_a_ value sees an apparent increase from 17.05 to 39.21 kJ mol^−1^ due to the high steric hindrance of large sulfolane-containing clusters (Fig. 2b). The resultant rate control of Sn^2+^ nucleation would benefit a smoother and more uniform deposition surface. Tafel plots were recorded to show the anti-corrosion property disparities of Sn metal in the two systems. In view of the confined activity of free H_2_O by the finely designed micelle structure, a slower corrosion tendency and a lower corrosion rate are expected in MCE. Its more positive corrosion potential (− 0.210 vs. − 0.216 V) and lower corrosion current density (4.3–2.3 mA cm^−2^) compared to the BE fully validates this distinctive merit (Fig. 2c). To visually highlight the advantageous property of micellar structure for retarding Sn corrosion, surface composition and morphology transition of Sn foils immersed in BE and MCE for 15 days were characterized through scanning electron microscopy (SEM) and X-ray diffraction (XRD). In BE, subject to prolonged H^+^ attack, metallic Sn suffers from oxidative dissolution that transforms initially smooth electrode interfaces into morphologically heterogeneous surfaces, accompanies by the appearance of microstructural defects consisting of tin oxide (SnO, PDF#41-1445) and stannous sulfate (SnSO_4_, PDF#81-0743) by-products (Fig. S13). Such morphological degradation amplifies the local charge accumulation during deposition/stripping cycles, thereby leading to uncontrolled bulk Sn growth that impairs electrochemical reversibility. In contrast, the surface morphology of the Sn foil immersed in MCE remains flat and no extra by-products are noticed (Fig. S14).

Although Sn exhibits a higher anti-corrosion property than Zn, another metal anode commonly utilized in aqueous batteries, HER may still compete with Sn deposition during long-term cycling in consideration of the strongly acidic environment [23]. Upon the occurrence of hydrogen evolution, porous, loose Sn deposition inevitably takes place, which is detrimental to the compact, smooth morphology. More importantly, it can further cause the detachment of Sn deposits that have limited contact with the substrate, exacerbating the annoying phenomenon of “Sn whisker” or “dead Sn.” Linear sweep voltammetry (LSV) from + 0.2 to − 0.9 V vs. SHE reveals the competitive dynamics between Sn^2+^ deposition and HER in the two systems. In both cases, a current peak ascribed to the diffusion limit of Sn^2+^ is noticed at − 0.23 V, accompanied by the presence of fine Sn crystals at the electrode surface. Further expansion of the potential range to − 0.67 V leads to a sharp/steep current escalation to > 1 A cm^−2^ in BE (Fig. 2d). Here, apart from Sn deposition, intense bubbling phenomenon is also detected due to the high reactivity of hydrated protons in acidic aqueous electrolyte. That is, the as-observed A-level current is attributed to the joint contribution of Sn plating and hydrogen evolution, but their intertwined interplay makes it difficult to distinguish their respective contribution. More importantly, the uneven deposition of Sn^2+^ ions would be exacerbated by the continuous evolution of hydrogen gas, as evidenced by the porous, “whisker-like” morphology of Sn deposits (Fig. S15a). Such uneven architecture substantially enlarges electrode–electrolyte contact area, in turn intensifying hydrogen evolution. In contrast, in MCE, the diffusion limit current peak at − 0.29 V is followed by a long, flat plateau from − 0.32 to − 0.72 V corresponding to the even, homogeneous Sn deposition without the interference of proton reduction. The suppression of hydrogen evolution leads to a lower deposition current (≈40 mA cm^−2^ at − 0.95 V where proton reduction dominates the reduction processes over Sn plating). Accordingly, the electrode surface remains compact and homogeneous after the LSV test (Fig. S15b). To enable a more intuitive analysis of the impact of micelle structure on HER, we conducted tests using pure 2 M H_2_SO_4_ and a micellar electrolyte composed of 2 M H_2_SO_4_ + SL. As shown in Fig. S16, in the 2 M H_2_SO_4_ + SL micellar electrolyte, the overpotential required to achieve a current of 40 mA cm^−2^ is significantly lower (2 M H_2_SO_4_: − 0.64 V vs. 2 M H_2_SO_4_ + SL: − 0.64 V). The increase in the hydrogen evolution overpotential can be ascribed to the sulfone-involved micelle structure that disrupts the original hydrogen bonding network of bulk H_2_O in the system [24], which limits the migration of H_2_O molecules and protons.

The very early stage of Sn crystal formation, the nucleation manner of Sn^2+^ ions in particular, is a decisive step regulating the plating kinetics and final morphology of Sn deposits. To decipher the underlying nucleation mechanism disparity of electroplating Sn in BE and MCE, we first compared the voltage profiles in the two systems. With the presence of sulfolane, the nucleation overpotential is increased from 5.2 to 13.3 mV (Fig. S17). As there is an inverse relationship between the overpotential (η) and the nucleation size (r) according to classical nucleation theory, the higher overpotential detected in MCE signifies a smaller nucleation size of Sn [25]. Cyclic voltammetry (CV) measurements were taken to investigate their respective nucleation behavior. As depicted in Fig. S18a, in BE, an apparent nucleation loop is observed in the initial cycle, but it disappears during the second cycle, which indicates the instantaneous nucleation manner of Sn. That is, the second-cycle Sn deposition undergoes rampant epitaxial growth instead of fresh nucleation, with new Sn layers expanding outward from the nuclei generated in the first cycle [13]. In MCE, distinct nucleation loops are observed in both cycles, signifying the progressive nucleation manner of Sn nucleus in the micellar electrolyte (Fig. S18b). Based on the high potential dependence of new phase formation during electrocrystallization and the direct response of current transients to interfacial reactions [26], chronoamperometry (CA) was employed to systematically investigate the nucleation and growth kinetics of Sn electrodeposition in the two systems (Fig. S19). The characteristic I–t curves were normalized into dimensionless forms of (*I*/*I*_m_)^2^ − (*t*/*t*_m_) (*I*_m_ and *t*_m_ correspond to the maximum peak current and its corresponding time, respectively) for quantitative analysis using the Scharifker–Hills diffusion-controlled nucleation model [27]. This theoretical framework classifies nucleation mechanisms into two distinct modes according to the dynamic activation mechanism of electrochemical active sites: when the substrate active site is momentarily saturated under overpotential driving, it forms spatially limited instantaneous nucleation. If the active site gradually activates with time and the nucleation rate is dominated by interfacial reactions, it exhibits progressive nucleation. The kinetic distinctions between these two modes are mathematically characterized by the following dimensionless equations (Eqs. 3 and 4):

3 $$\left(\frac{I}{{I}_{\mathrm{m}}}\right)^{2}=\frac{1.9542}{\left(\frac{{t}}{{{t}}_{\mathrm{m}}}\right)}{{{1-\exp}\left[{-1.2564}\left(\frac{{t}}{{{t}}_{\mathrm{m}}}\right)\right]}^{2} }\text{(instantaneous nucleation)}$$

4 $$\left(\frac{I}{{I}_{\mathrm{m}}}\right)^{2}=\frac{1.2254}{\left(\frac{{t}}{{{t}}_{\mathrm{m}}}\right)} \left\{1-\exp\left[-2.3367\left(\frac{t}{t_{\mathrm{m}}}\right)^{2}\right]\right\}^{2} (\text{progressive nucleation})$$

As shown in Fig. 2e, in BE, the normalized dimensionless curves at potentials of − 0.33 to − 0.43 V vs. SHE aligns well with the instantaneous nucleation theoretical model (simulated according to Eq. 3, indicating instantaneous saturation of nuclei during the initial stage, followed by growth dominated by preexisting nuclei). This behavior likely originates from the coupled effects of fast Sn^2+^ ion mass transport and interfacial reduction. In stark contrast, the normalized CA curve in the MCE system (Fig. 2f) consistently matches the progressive nucleation model (simulated according to Eq. 4), pinpointing the capability of the micellar network to switch the nucleation pathway of Sn. That is, the coordinated slow release of Sn^2+^ ions substantially relieves the local concentration gradient at the electrode/electrolyte interface, which promotes uniform spatial distribution of nuclei, where nucleation sites are gradually activated alongside concurrent nucleus growth. Such time-dependent progressive activation of nucleation sites is anticipated to alleviate uncontrolled Sn growth and thereby smoothen the Sn deposition morphology. When the applied polarization voltage is amplified, the current density in the BE rapidly increases within 100 s, indicating the presence of a sustained and uncontrolled 2D diffusion process (Fig. S20). In contrast, the MCE exhibits a 2D nucleation phase lasting only ~ 7 s, followed by a long-term stable 3D dense diffusion process. This demonstrates that the confining effect of the micelle structure effectively modifies the disordered diffusion of Sn^2+^, thereby controlling its nucleation mechanism and yielding a dense and smooth deposited layer.

To intuitively present how the nucleation mechanism difference affects the Sn deposition behavior, we then calculated the full width at half maximum (FWHM) of the (211) facet in BE and MCE according to the Scherrer equation. The large expansion of FWHM from 0.20 to 0.31 again validates that the progressive nucleation guided by the micelle structure favors a smaller average crystallite size of the Sn deposits [12] (Fig. 2g). The morphology changes of the Sn deposits on the Cu substrate in the two systems were then analyzed by SEM to corroborate the above explanation. In BE, Sn distributes scatteringly on Cu, with the appearance of long Sn whiskers (> 500 μm) that can easily derive to “dead Sn” (Fig. 2h). In sharp contrast, in MCE, Sn is uniformly distributed with smaller particle sizes and no whisker or ‘dead Sn’ is noticed in the millimeter-scale region (Fig. 2i). It is more clearly demonstrated the superior ability of MCE to minimize the deposition size of Sn deposits, contributed by the nucleation manner transition from instantaneous to progressive, which is essential to achieve reversible Sn deposition/dissolution free of the interference of “dead Sn” over extended periods. Building upon above discussions, we summarize the reaction mechanisms in the two electrolyte systems as follows: In the BE system, rapid reaction kinetics drive swift Sn^2+^ reduction, leading to instantaneous nuclei formation on the substrate within short timeframes. As the energy barrier for growth becomes lower than that for nucleation, the system preferentially continues deposition on existing nuclei rather than generating new nucleation sites, ultimately forming large polyhedral particles or whisker-like structures (Fig. 2j). On the contrary, in the MCE system, the micelle confinement effect guides the controlled release of Sn^2+^ under an external electric field, which regulates the ordered reduction of Sn^2+^ by slowing down the diffusion and reaction kinetics. This controlled process induces a progressive nucleation mode during electrodeposition, where uniformly distributed Sn nuclei guide subsequent growth pathways, ultimately producing smooth Sn deposits with homogeneous morphology (Fig. 2k).

**Fig. 3:**
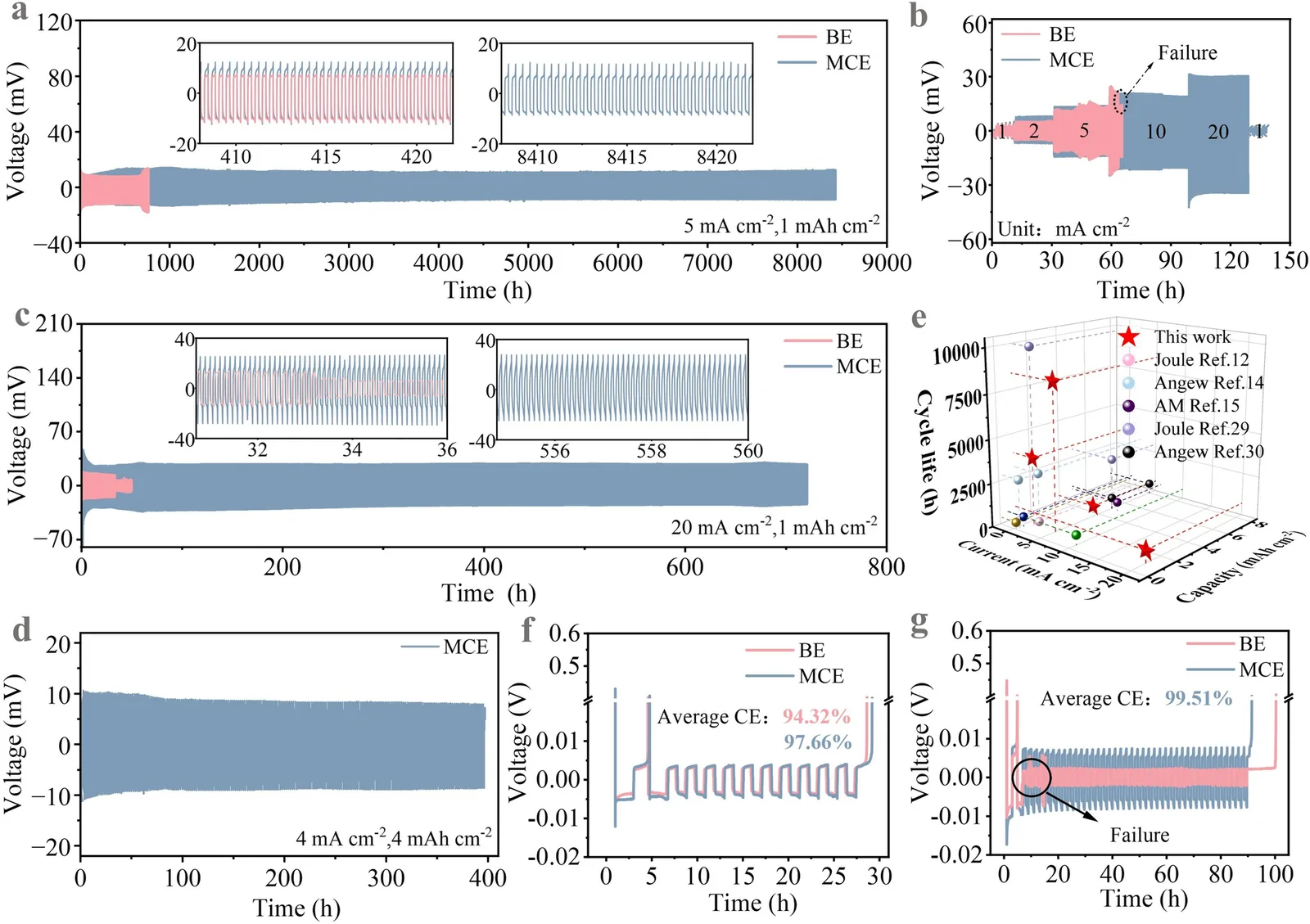
**a** Cycling performance of Sn||Sn symmetric cells in BE and MCE at 5 mA cm^−2^, 1 mAh cm^−2^. **b** Rate performance of Sn anode in BE and MCE. **c** Galvanostatic Sn plating/stripping curves of Sn||Sn symmetric cells at 20 mA cm^−2^, 1 mAh cm^−2^ in BE and MCE and **d** 4 mA cm^−2^, 4 mAh cm^−2^ in MCE. **e** Electrochemical performance of Sn||Sn with MCE in comparison with recently reported references. Aurbach method to measure the average CE with **f** 1 mA cm^−2^, 2 mAh cm^−2^ for 10 cycles and **g** 2 mA cm^−2^, 4 mAh cm^−2^ for 40 cycles

The above experimental results and theoretical analysis have amply demonstrated the promising potential of micelle electrolyte in improving the Sn chemistry reversibility by virtue of the following two aspects: (i) enabling a progressive nucleation mechanism through kinetic retardation of Sn^2+^ deposition and (ii) suppressing the reactivity of hydrated protons by restructuring hydrogen bonding networks. Then, Sn||Sn symmetric coin cells were assembled in BE and MCE to evaluate the influence of the micelle electrolyte on the Sn plating/stripping courses. When operated at 1 mA cm^−2^ and 1 mAh cm^−2^, Sn anode can be stably cycled over 4000 h in MCE (Fig. S10). More encouraging, an ultralong cycle duration of more than 8400 h is noticed at 5 mA cm^−2^ and 1 mAh cm^−2^ (Fig. 3a). However, Sn exhibits inferior cycling stability in BE and suddenly encounters short circuits at 710 h. To explore the rate capability in different electrolytes, Sn||Sn cells were cycled at different current densities ranging from 1 to 20 mA cm^−2^ with a constant areal capacity of 1 mAh cm^−2^ (Fig. 3b). Sn fails to sustain stable Sn deposition/exfoliation as the current density attains 10 mA cm^−2^ in BE, whereas MCE maintains stable operation even at 20 mA cm^−2^. The enlarged views of 1 and 20 mA cm^−2^ are shown in Fig. S21. Both figures exhibit stable voltage changes, confirming that no short circuit occurred. The as-observed rate performance superiority of MCE over BE is also witnessed from Sn||Cu symmetric coin cells (Figs. S22 and S23). Generally, high current densities exacerbate the interfacial concentration gradient due to the accelerated depletion of Sn^2+^ at the electrode/electrolyte interface, which exacerbates the occurrence of undesired “Sn whiskers” or “dead Sn” that poison the anode life span. As expected, even at a high current density of 20 mA cm^−2^ where Sn can hardly work correctly in BE, the symmetric cell with MCE exhibits a long cycle life of over 700 h, highlighting the positive role of the progressive nucleation manner in promoting reversible Sn chemistry at harsh conditions (Fig. 3c).

In previous studies, the longevity of Sn anode is generally achieved under a shallow utilization rate of Sn [12–14, 28]. This phenomenon may originate from the inability to modify subsequent nucleation mechanisms despite improvements in the initial Sn particle morphology, resulting in insufficient densification of the deposited layer. In turn, the formation of a non-dense deposition layer significantly increases the specific surface area of Sn, thereby intensifying the HER and accelerating the accumulation of “dead Sn.” How to realize a high specific capacity for Sn deposition remains challenging. In our case, even when the areal capacity goes up to 4 mAh cm^−2^, highly reversible Sn plating/stripping process can be still maintained for nearly 380 h (Fig. 3d), fully demonstrating the critical role of the micellar electrolyte-mediated progressive nucleation mechanism in achieving dense Sn deposition. To prominently highlight the superiority of our work, cycling life span, current density and capacity were selected as critical aspects for comparing the Sn plating/stripping performance in micelle electrolyte with the state-of-the-art one reported in the literature [12, 14, 15, 29, 30]. Clearly, our work tops the list, integrating exceptional current density tolerance, high areal capacity and extended cycling stability (Fig. 3e). To more accurately evaluate the reversible Sn utilization and quantify capacity fade from side reactions, Sn||Cu asymmetric cells were assembled in BE and MCE to assess the CE, a key parameter representing the electrochemical reversibility. The Sn||Cu cell with MCE maintains an exceptionally high average CE of 99.97% over 5300 cycles at 5 mA cm^−2^, 1 mAh cm^−2^, whereas the counterpart with BE suffers from rapid failure after just 46 cycles, seeing a lower average CE of 98.91% (Fig. S24). To further verify the advantage of MCE in improving CE, Aurbach method was employed to measure the CE of the Sn||Cu system [31]. In this approach, controlled Sn deposition (*Q*_p_) is first executed on a pristine Cu current collector, followed by complete anodic stripping under identical current density conditions. The average CE is verified through repeated stripping/deposition under a low current density (1 or 2 mA cm^−2^) and 50% utilization rate of Sn (*Q*_c_ = *Q*_p_ × 50%). Finally, the Sn deposited on the Cu surface is fully stripped (*Q*_s_) to calculate the average CE. After a short cycle of 10 repetitions with *Q*_p_ of 2 mAh and current density of 1 mA cm^−2^, MCE elevates the average CE from 94.32% to 97.66% (Fig. 3f). Moreover, when *Q*_p_ is increased to 4 mAh and the current density is 2 mA cm^−2^, where the cell with BE can hardly work, the CE with MCE reaches 99.51% after 40 cycles (Fig. 3g). Such an outstanding improvement of CE demonstrates the significant role of MCE in improving the stability and reversibility of Sn anodes.

**Fig. 4:**
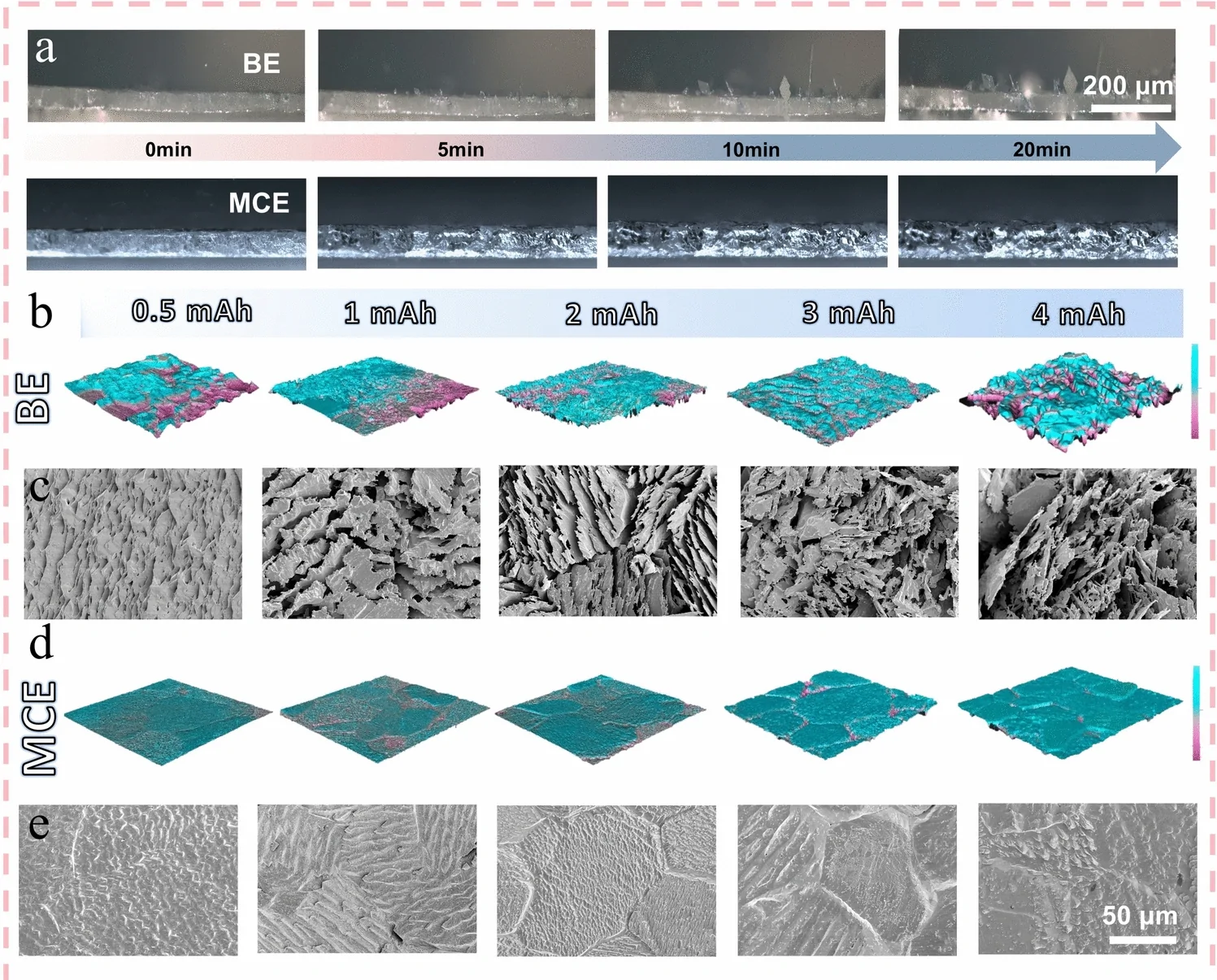
**a** In situ monitoring of Sn growth by optical microscopy in BE and MCE at 10 mA cm^−2^. CLSM and SEM images of Sn anode with **b-c** BE and **d-e** MCE at varied deposition capacities from 0.5 mAh cm^−2^ to 4 mAh cm^−2^, the color bar ranges from 10.76 μm (violet) to −13.64 μm (blue)

To unravel the underlying mechanism behind the prolonged cycle life of Sn in MCE, a series of morphology characterizations were conducted to visually compare Sn deposition behaviors in the two electrolytes. In situ optical microscopy observations were first employed to track the surface transition of metallic Sn anode within 20 min from a macroscopic perspective. As depicted in Fig. 4a, after only 5 min deposition at 10 mA cm^−2^, visible protrusions are clearly detected on Sn surface in BE. With the proceeding of electroplating, these protrusions become more and more prominent and finally form large “standing” particles having limited contact with the substrate. These particles are prone to detach and derive to “dead Sn,” which ultimately leads to rapid electrochemical performance decay. In sharp contrast, thanks to homogenous Sn^2+^ deposition mediated by micellar structure in MCE, the flat, smooth and compact morphology of Sn anode is constantly kept throughout electrochemical plating process. 3D confocal laser scanning microscopy (CLSM) was then utilized to observe the microscopic morphology differences of Sn deposited in BE and MCE. As shown in Fig. 4b, d, after 50 cycles at different deposition capacities, the morphology in the BE system gradually becomes rougher and more porous with increasing deposited surface area. In MCE, the surface of Sn foil remains smooth and flat from 0.5 to 4 mAh cm^−2^. Microscopic surface characterization by SEM in Fig. 4c, e comes to the same conclusion on the positive role of MCE in smoothening Sn deposition at varied capacities. As expected, in BE, Sn anode suffers from progressive structural deterioration with the escalation of deposition areal capacity. The surface morphology experiences gradual transition from loosely stacked lamellar structures to porous networks adorned with dendritic serrations. At 4 mAh cm^−2^, irregular “Sn whiskers” with an ultralong length greater than 500 μm are noticed (Fig. S25). The derivation of “Sn whisker” to “dead Sn” further creates preferential sites for subsequent irregular deposition, accelerating surface roughening through self-perpetuating deterioration. Remarkably, in the MCE system, the anode surface maintains a relatively flat surface without large Sn particles or detectable separation scars from 0.5 to 4 mAh cm^−2^. To verify whether MCE affects crystal planes, we conducted XRD testing on tin foil with different numbers of deposition cycles. However, in our system, the deposited Sn exhibits no particular orientation, maintaining its isotropic metallic properties as it grows randomly across all crystal planes (Fig. S26). The as-observed morphology modulation of Sn deposits in MCE should be ascribed to the self-assembly of micellar structure endowed by the amphoteric nature of sulfolane. That is, the encapsulation of Sn^2+^ ions in nanoscale micelles can slow down the Sn deposition kinetics by activating the very initial progressive nucleation manner of Sn^2+^. The minimized size of Sn deposits accompanied by inhibited HER conjointly contributes to uniform Sn deposition, even under high-capacity conditions. To validate this concept, DMSO, another amphoteric small organic molecule containing both hydrophilic and hydrophobic groups, is introduced into BE to see its impact on the plating/stripping behavior of Sn. As shown in Fig. S27, prominent Tyndall effect is observed in DMSO-containing electrolyte, signifying the presence of the micellar structure. In addition, like sulfolane, it is also capable of smoothening Sn^2+^ deposition, even at 4 mAh cm^−2^ (Fig. S28). It is thus fully demonstrated our assumption of the micellar electrolyte structure for improving Sn chemistry, which can also be extended to other aqueous systems sharing similar fundamentals.

**Fig. 5:**
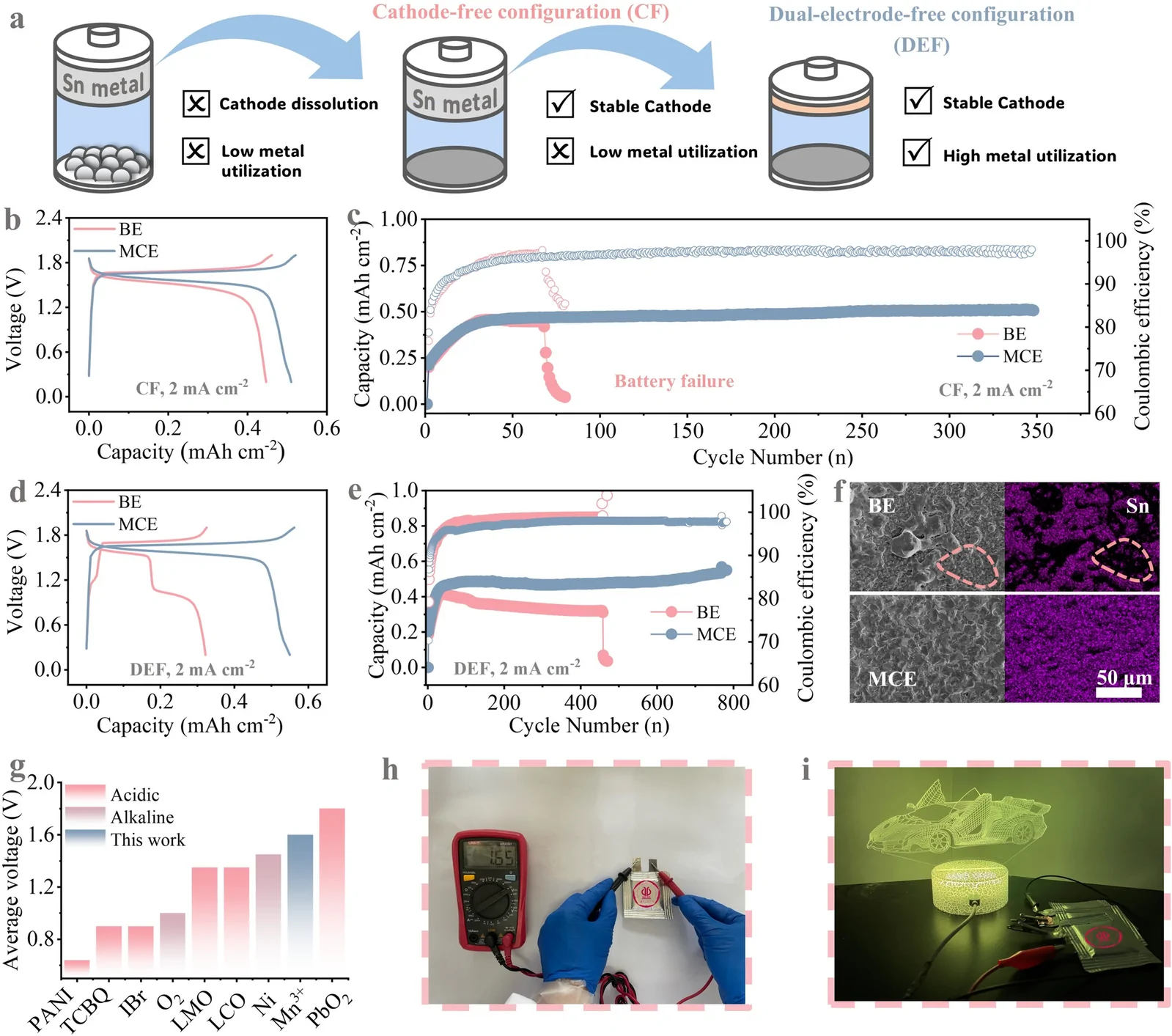
**a** Schematic illustration of the conventional battery configuration, cathode-free configuration (CF) and dual-electrode-free configuration (DEF). **b** GCD curves and **c** cycling stability of **b**–**c** CF and **d**–**e** DEF at 2 mA cm^−2^. **f** SEM images and elemental mapping of Sn deposited on Cu substrate in BE and MCE. **g** Comparison of average voltage of various cathodes reported in Sn-based aqueous batteries. **h** Voltage of pouch battery (3 cm × 3 cm) after charging and **i** Four pouch cells in series illuminating supercar

In view of the excellent contribution of MCE to stabilize Sn deposition/dissolution chemistry, Sn||Mn full batteries were further constructed to stress its feasibility for practical applications. Distinct from conventional aqueous batteries, a cathode-free (CF) configuration is selected to avoid the capacity decay issue initiated by the dissolution of conventional manganese-based cathodes [32] (Fig. 5a). That is, Sn anode is coupled with graphite paper (current collector) in BE (denoted as Sn|BE|Mn) and MCE (denoted as Sn|MCE|Mn) supplemented with 1.6 M MnSO_4_. The CV curves of both batteries (Fig. S29) deliver comparable redox peaks at 1.7/1.6 V assigned to the conversion between Mn^3+^ and Mn^2+^. Theoretically, Mn^2+^/MnO_2_ conversion also exhibits thermodynamic feasibility. To investigate whether MnO_2_ deposition on the cathode in our system, we performed XRD analysis on fully charged cathode electrodes after 50 cycles (Fig. S30). The results reveal that the spectra of electrodes cycled in both electrolytes matched those of the initial carbon paper, with no MnO_2_ peaks detected. Therefore, the overall reaction of Sn||Mn batteries is given as follows [33]:

$${\mathrm{2M}}{{\mathrm{n}}^{{2} + }} + {\mathrm{S}}{{\mathrm{n}}^{{2} + }} \rightleftharpoons {\mathrm{2M}}{{\mathrm{n}}^{{3} + }} + {\mathrm{Sn}}$$

Noticeably, compared to Sn|BE|Mn, Sn|MCE|Mn exhibits smaller polarization and higher peak current, implying that the presence of sulfone also benefits the Mn^3+^/Mn^2+^ conversion kinetics. Density functional theory calculations were employed to investigate the interactions between SL and Mn^3+^ that constitute the micelle structure. As shown in Fig. S31, we calculated the binding energies between Mn^3+^ ions and sulfolane/H_2_O. The results indicate that the binding energy between sulfolane and Mn^3+^ is significantly stronger than that with H_2_O (− 2.15 vs. − 2.68 eV). This computational result strongly suggests that sulfolane can selectively stabilize Mn^3+^ ions in the electrolyte. Correspondingly, in galvanostatic charge–discharge (GCD) curves, the two devices deliver similar plateaus around 1.6 V at 2 mA cm^−2^, but Sn|MCE|Mn presents lower polarization and higher capacity (Fig. 5b). In terms of the cycling durability, benefiting from the improved Sn chemistry by micellar electrolyte structure, the battery life span is extended from 75 to 350 cycles in MCE with a capacity of ≈0.5 mAh cm^−2^ (Fig. 5c). In this case, the utilization efficiency of Sn anode is quite low since only a tiny fraction of Sn foil (50 μm) functions as active materials. Leveraging the strong affinity between Cu and Sn, a dual-electrode-free (DEF) Sn||Mn full battery was then assembled by utilizing Cu as the anode current collector. As shown in Figs. 5d and S32, the DEF batteries with MCE maintain a similar flat plateau at approximately 1.6 V. Owing to the excellent polarization voltage of Sn||Mn, both battery systems initially exhibit high energy efficiency (Fig. S33). However, beyond 200 cycles, the BE system suffers a significant efficiency drop to 78.59%, which is attributed to severe side reactions. Concurrently, an additional voltage plateau emerges around 1.1 V alongside the original 1.6 V plateau (Fig. S34). This new plateau is confirmed to correspond to copper deposition/dissolution, as verified by a control experiment with Cu^2+^ containing electrolyte (Fig. S35). In contrast, the MCE system maintains remarkable stability over 790 cycles, preserving 83.81% energy efficiency, thanks to effective anode protection and suppression of Mn^3+^ disproportionation by the micelle. The involvement of Cu in electrochemical reaction can be triggered by the uneven deposition of Sn in BE, which leaves the exposure of numerous ‘Cu hot spots’ that suffer from electrolyte corrosion (Fig. S36). To consolidate this viewpoint, we further conducted the elemental mapping of cycled Cu surfaces by SEM (Figs. 5f and S37). In accordance to our prediction, uniform Sn deposition in MCE results in full coverage of Cu by freshly deposited Sn. Yet, scattered Cu sites are directly exposed to the strong acidic electrolyte, which well explains the gradual electrochemical dissolution of Cu observed in BE. Such irreversible consumption of Cu current collector in BE significantly compromises the cycling stability of the battery (battery failure after only 450 cycles, Fig. 5e). As the electrode structure is well maintained, the DEF battery can endure stable cycling for 790 cycles in MCE, along with a competitive average discharge voltages in contrast to Sn-based batteries in both acidic and alkaline electrolyte systems (Fig. 5g). Finally, pouch cell with a dimension of 3 cm × 3 cm was further assembled to assess the practicality of Sn||Mn batteries assembled in MCE (Fig. 5h). As illustrated in Fig. 5i, three tandem pouch cells can power a 4.5 V modern supercar LED light.

## Conclusions

In summary, we leveraged the hydrophilic and hydrophobic properties of sulfolane molecules (has the dual capability of solvating a Sn^2+^ salt and interacting with H_2_O molecules) to self-assemble in the electrolyte, forming a novel micellar electrolyte, and successfully achieved a highly reversible aqueous acidic Sn anode. Theoretical calculations and experiments show that micellized sulfolane forms a strong coordination bond with Sn^2+^, encapsulating it within the micelle structure. The structure of the micellar framework creates a significant kinetic barrier through steric hindrance, while its strong coordination with Sn^2+^ ions ensures a gradual release of the ions, which realizes the transition from an instantaneous nucleation deposition mode to a progressive nucleation deposition mode. In addition, the micellar structure reconstructs the hydrogen bonding network within the system and disrupts the rapid proton transport pathway, which greatly suppress HER. Consequently, the life span of the Sn||Sn cell is significantly extended from 710 to 8400 h at 5 mA cm⁻^2^ and 1 mAh cm⁻^2^. Even at a high current of 20 mA cm^−2^, it can still cycle stably for more than 700 h. Furthermore, the assembled cathode-free Sn||Mn battery achieved a high voltage of 1.6 V and maintained an average capacity of 0.48 mAh cm^−2^ over 350 cycles. Even under the harsh condition of dual-electrode-free, the system achieves over 790 stable cycles while maintaining a capacity of 0.5 mAh cm^−2^. This work is expected to shed light on understanding Sn chemistry in aqueous environment and thereby give new train of thoughts for the construction of advanced aqueous Sn batteries.

## Supplementary Information

Below is the link to the electronic supplementary material.

Supplementary file1 (DOCX 17064 kb)
