# Supplementary material for: Activating Progressive Sn2+ Nucleation by Micellar Structure Electrolyte for Dead-Sn-Free Aqueous Batteries
Source: Nanomicro Lett. 2026 Feb 3;18:234. doi: 10.1007/s40820-026-02070-6 (PMC12868521; doi:10.1007/s40820-026-02070-6)
Supplement: Supplementary file 1 — Supplementary file1 (DOCX 17064 kb) [file 40820_2026_2070_MOESM1_ESM.docx]

Supporting Information for

**Activating Progressive Sn^2+^ Nucleation by Micellar Structure Electrolyte for Dead-Sn-Free Aqueous Batteries**

Xiaojia Lan^1,2^, Zhaoyu Zhang^1,2^, Yuekai Lin^1,2^, Wencheng Du^1,2^, Yufei Zhang^1,2^, Minghui Ye^1,2^, Zhipeng Wen^1,2^, Yongchao Tang^1,2^, Xiaoqing Liu^1,2,^*, and Cheng Chao Li^1,2,^*

^1^ School of Chemical Engineering and Light Industry, Guangdong University of Technology, Guangzhou 510006, People’s Republic of China

^2^ Guangdong Provincial Laboratory of Chemistry and Fine Chemical Engineering Jieyang Center, Jieyang 515200, P. R. China

*Corresponding authors. E-mail: [licc@gdut.edu.cn](mailto:licc@gdut.edu.cn) (Cheng Chao Li); [liu@gdut.edu.cn](mailto:liu@gdut.edu.cn) (Xiaoqing Liu)

**S1** **Experimental Section**

**S1.1 Material Characterizations**

Dynamic light scattering was measured by utilizing a zeta potential analyzer (Zetasizer NANO ZS, Malvern, UK). FT-IR spectra were obtained by an FT-IR spectrometer (Nicolet iS5, Thermo Scientific). Raman spectra were collected by using a Raman spectrometer (LabRAM HR Evolution). NMR spectra were provided by NMR spectrometer (JNM-ECZ500R/S1, Japan). SEM images were taken by field emission scanning electron microscope (SU8220, Hitach, Japan). XRD patterns obtained by X-ray diffractometer (D8 VENTURE, Bruker, Germany). In-situ optical microscopy images were gained by a DMM-900C metallographic microscope (Caikon Optical Instrument Co., Ltd, Shanghai). CLSM images were acquired by confocal laser scanning microscope (OLS4100, Olympus, Japan).

**S1.2 Simulation methods**

The density functional theory calculations for water, salt, and corresponding Sn^2+^ complexes were performed in the Gaussian. The pictorial representations of the molecular structures were prepared using the Visual Molecular Dynamics and Multiwfn software. An implicit solvation model based on density was used to consider solvent effects. The binding energy (E_Binding_) of Sn^2+^ complexes were calculated using the following equation:

𝐸*_Binding_* = 𝐸_Sn_^2+^_-complexe_ ‒ 𝐸_complexe_ ‒ 𝐸*_Sn_^2+^*

where E_Sn_^2+^_-complex_ represents the total energy of the complex interacting with Sn^2+^, E_complex_ is the energy of the complex, and E_Sn_^2+^ is the energy of a single Sn^2+^.

Atomistic molecular dynamics simulations have been performed in the GROMACS [S1] (version 2022.6) simulation package using the CHARMM 36 force field [S2] and the TIP3P water model. The parameters for Sn^2+^ ion used the parameters of the UFF force field. Two systems were simulated, one containing 260 H_2_SO_4_, and 26 SnSO_4_ and 6669 water molecules; the other containing additional 136 sulfolane molecules and water were reduced to 5878 molecules. The molecules were first randomly placed in cubic boxes of around 6 nm and water was used to solvate the whole system. Then, the ions were inserted through random replacement of water molecules. After thousands of steps of energy minimization, the systems were equilibrated for 5 ns under the NPT ensemble and followed the production runs of 10 ns under the canonic ensemble. An integration time-step of 1 fs was used and the temperature was coupled to 298 K using the Nose-Hoover method and a cutoff scheme of 1.2 nm was used for the non-bonded interactions, and the Particle Mesh Ewald method [S3] with a fourierspacing of 0.1 nm was applied for the long range electrostatic interactions. All covalent bonds with hydrogen atoms were constraint using the LINCS algorithm [S4]. The presence of hydrogen bonds was calculated using the common criteria so that the maximum angle of the hydrogen-donor-acceptor was 30 degrees, and the distance between the acceptor and donor atoms was less than 0.35 nm.

**Supplementary Figures and Tables**


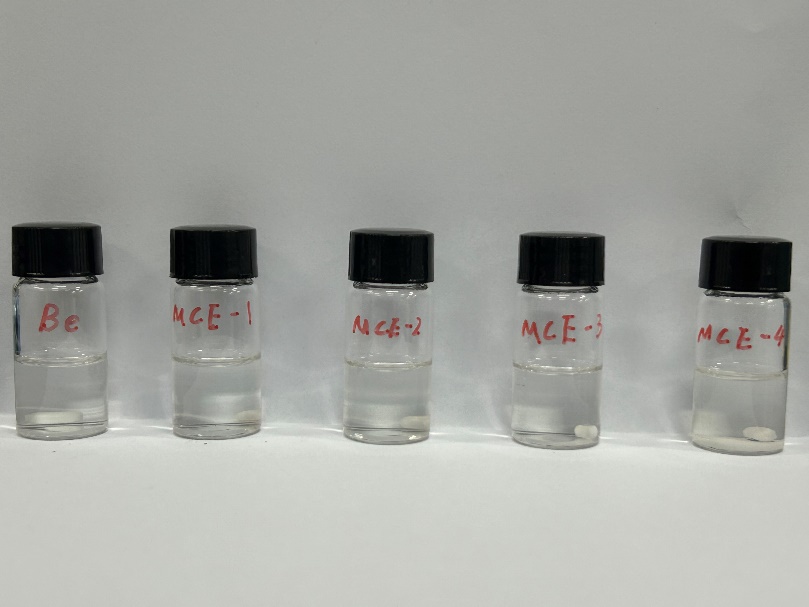


**Fig. S1** Optical photographs of various electrolytes containing different contents of sulfone

**Fig. S2** The electrostatic potential of sulfone and water

**Fig. S3** Binding energy of H_2_O-H_2_O and Sulfone-H_2_O

**Fig. S4** DLS analysis of the micelle particle size in MCE-1, MCE-2 and MCE-3

**Fig. S5** (**a**) The Raman spectra of different electrolytes (BE, MCE-1, MCE-2, MCE-3) and (**b**) The local enlargement signal attributed to SO_4_^2-^

**Fig. S6** (**a**) The fitted O-H stretching vibration represents water molecules with strong (pale Chestnut), mid (pink) and weak (blue) H-bond and (**b**) The proportion of strong H-bond water varied by the SL concentration


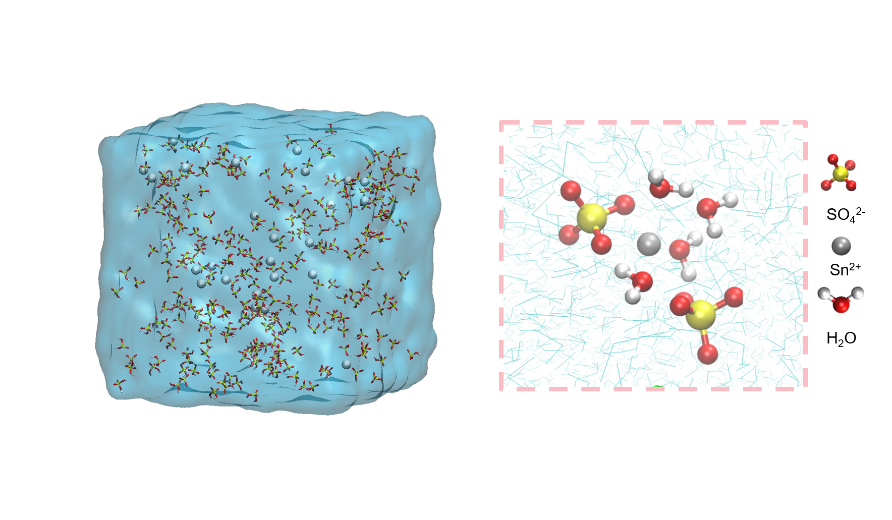


**Fig. S7** 3D snapshots of MD simulations for BE with corresponding schematic diagrams of Sn^2+^ solvation structures

**Fig. S8** RDF of Sn-O (H_2_O), Sn-O (SO_4_^2-^) and the corresponding coordination numbers in BE

**Fig. S9** Mean square displacement (MSD) curves for BE and MCE

**Fig. S10** The cycling performance of Sn||Sn at 1 mA cm^-2^ and 1 mAh cm^-2^ in different electrolytes

**Fig. S11** DLS analysis of the micelle particle size in MCE and BE after 100 cycles

**Fig. S12** Variable temperature electrochemical impedance spectroscopy (EIS) results of (**a**) BE and (**b**) MCE

**Fig. S13** SEM images of Sn anodes after immersion in (**a**) BE and (**b**) MCE for 15 days

**Fig. S14** (**a**) complete and (**b-c**) partial enlarged XRD patterns after immersion in BE and MCE for 15 days

**Fig. S15** Optical photos of Sn deposited on Cu substrate in (**a**) BE and (**b**) MCE in after LSV test

**Fig. S16** The LSV curves of 2 M H_2_SO_4_ and 2 M H_2_SO_4_ + SL

**Fig. S17** The voltage–time curves during Sn nucleation at 1 mA cm^‒2^

**Fig. S18** CV curves of Sn^2+^ deposition/dissolution curves on the Cu substrates in (**a**) BE and (**b**) MCE, insect: the enlargement of the nucleation loop

**Fig. S19** CA curves of (**a**) BE and (**b**) MCE with an applied potential of −0.33 V, −0.38 V, and −0.43 V

Here, glassy carbon electrodes (GCE) were selected as the working electrodes for nucleation studies due to their inherent advantages aligning closely with the core requirements for characterizing nucleation behavior. First, the GCE exhibits excellent chemical inertness in electrolytes, preventing unexpected side reactions that could interfere with signals from tin nucleation and growth. Second, its smooth, uniform surface provides evenly distributed nucleation sites, minimizing the impact of surface defects or impurities on nucleation kinetics. This is crucial for accurately distinguishing transient nucleation from progressive nucleation patterns through CA curve fitting. Additionally, the GCE's high conductivity and low background current characteristics enable sensitive detection of minute current changes generated during initial nucleation.

**Fig. S20** CA curves in BE and MCE under −100 mV

**Fig. S21** Magnified view at (**a**) 1 mA cm^-2^ and (**b**) 20 mA cm^-2^ during magnification testing

**Fig. S22** The rate performance of Sn||Cu half cells in (**a**) BE and (**b**) MCE

**Fig. S23** Coulombic efficiency corresponding to the rate performance of the Sn||Cu half-cell

It is evident that MCE maintains stable operation across all current levels, with CE exhibiting a positive correlation as current gradually increases (~99.5%). In contrast, BE shows severe fluctuations in CE after only a few cycles, likely due to significant dead Sn shedding causing battery failure.

**Fig. S24** (**a**) CE of the Sn stripping/plating in BE and MCE at 5 mA cm^‒2^, 1 mAh cm^‒2^ and (**b**) corresponding capacity-voltage curves from the 1^st^ to 5000^th^ cycle for MCE


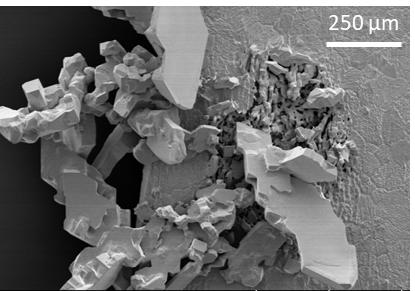


**Fig. S25** SEM images of Sn anodes cycled in BE at 4 mAh cm^-2^

**Fig. S26** XRD patterns of tin foil after different numbers of cycles in two electrolytes


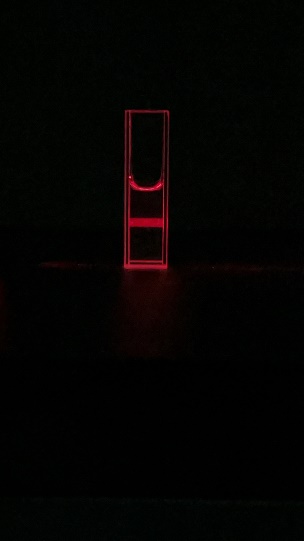


**Fig. S27** Tyndall effect of BE supplemented with 10% v/v DMSO


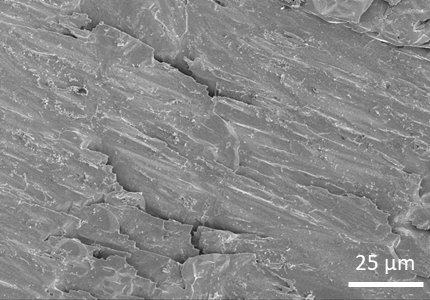


**Fig. S28** SEM images of Sn anodes cycled in BE supplemented with 10% v/v DMSO at 4 mAh cm^-2^

**Fig. S29** CV curves of Sn|BE|Mn and Sn|MCE|Mn at a scan rate of 1 mV s^-1^ with CF

**Fig. S30** XRD pattern of the cathode electrode after 50 cycles

**Fig. S31** Binding energy of H_2_O-Mn^3+^ and SL-Mn^3+^

**Fig. S32** CV curves of Sn|BE|Mn and Sn|MCE|Mn at a scan rate of 1 mV s^-1^ with DEF

**Fig. S33** Under DEF, the capacity-voltage curves of BE and MCE at different cycle numbers

**Fig. S34** Energy efficiency comparison in long-tern cycles


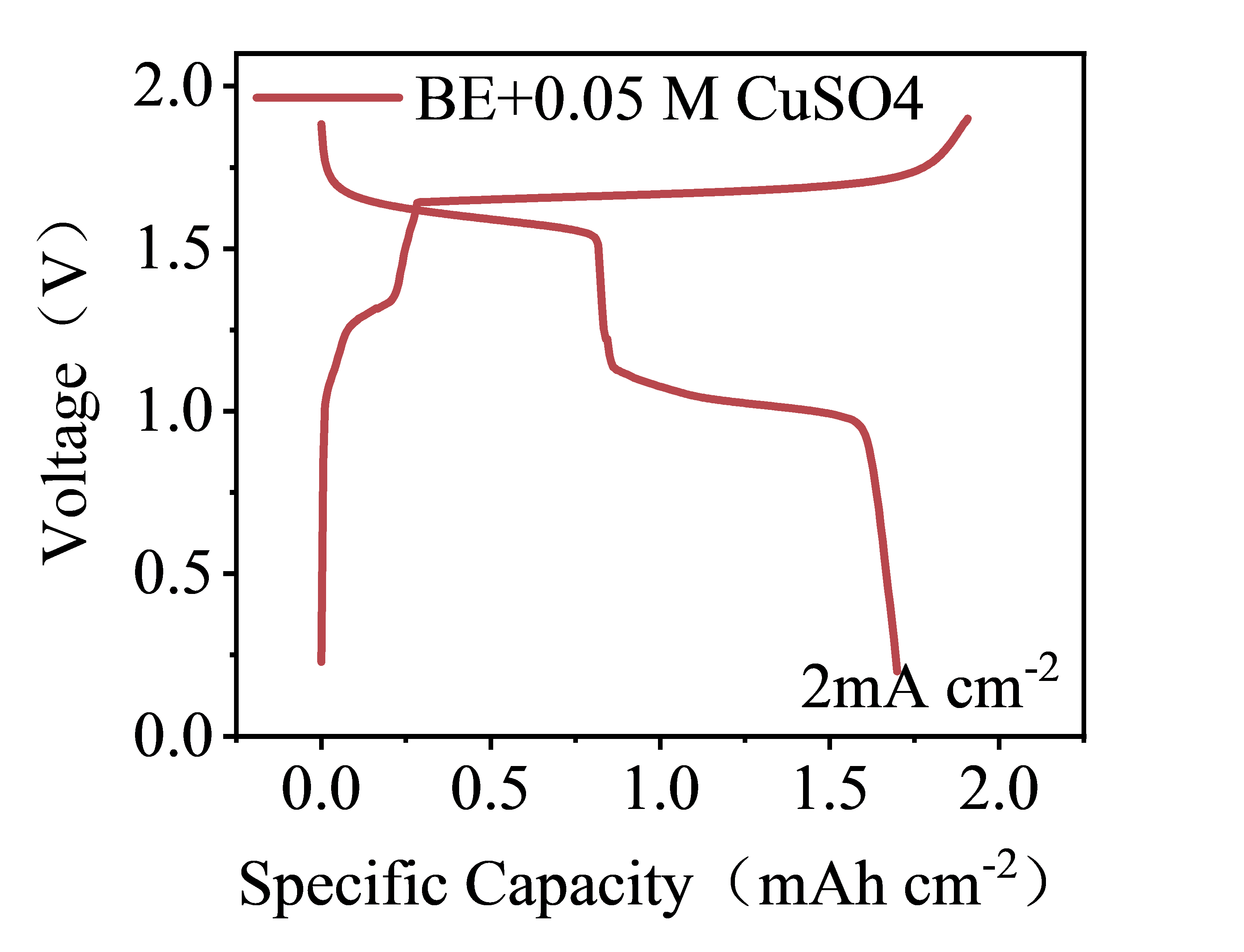


**Fig. S35** The capacity-voltage curves of BE+0.05 M CuSO_4_


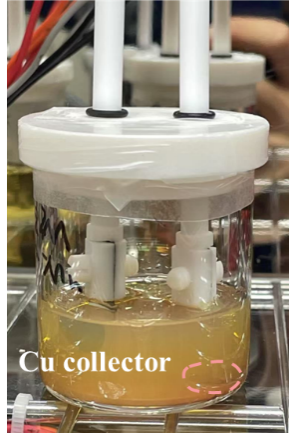


**Fig. S36** Optical photos of BE battery after failure under DEF

**Fig. S37** Under DEF, SEM and elemental mapping of Cu current collector surface after cyclic charging

**Supplementary References**

1. B. Hess, C. Kutzner, D. van der Spoel, E. Lindahl, GROMACS 4:   algorithms for highly efficient, load-balanced, and scalable molecular simulation. J. Chem. Theory Comput. **4**(3), 435–447 (2008). <https://doi.org/10.1021/ct700301q>
2. R.B. Best, X. Zhu, J. Shim, P.E.M. Lopes, J. Mittal et al., Optimization of the additive CHARMM all-atom protein force field targeting improved sampling of the backbone ϕ, ψ and side-chain χ1 and χ2 dihedral angles. J. Chem. Theory Comput. **8**(9), 3257–3273 (2012). <https://doi.org/10.1021/ct300400x>
3. U. Essmann, L. Perera, M.L. Berkowitz, T. Darden, H. Lee et al., A smooth particle mesh Ewald method. J. Chem. Phys. **103**(19), 8577–8593 (1995). <https://doi.org/10.1063/1.470117>
4. B. Hess, H. Bekker, H.J.C. Berendsen, J.G.E.M. Fraaije, LINCS: a linear constraint solver for molecular simulations. J. Comput. Chem. **18**(12), 1463–1472 (1997). [https://doi.org/10.1002/(sici)1096-987x(199709)18:121463::aid-jcc4>3.3.co;2-l](https://doi.org/10.1002/(sici)1096-987x(199709)18:121463::aid-jcc4%3e3.3.co;2-l)
